# Mito-Tempo improves acrosome integrity of frozen-thawed epididymal spermatozoa in tomcats

**DOI:** 10.3389/fvets.2023.1170347

**Published:** 2023-08-07

**Authors:** Hiba Ali Hassan, Penelope Banchi, Guillaume Domain, Leen Vanderheyden, Sylwia Prochowska, Wojciech Nizański, Ann Van Soom

**Affiliations:** ^1^Reproductive Biology Unit, Faculty of Veterinary Medicine, Department of Internal Medicine, Reproduction and Population Medicine, Ghent University, Ghent, Merelbeke, Belgium; ^2^Department of Veterinary Sciences, Faculty of Veterinary Medicine, University of Turin, Grugliasco, Italy; ^3^Department of Reproduction and Clinic of Farm Animals, Faculty of Veterinary Medicine, Wrocław University of Environmental and Life Sciences, Wrocław, Poland

**Keywords:** cat, epididymal spermatozoa, antioxidant, Mito-Tempo, reactive oxygen species

## Abstract

**Introduction:**

In tomcats, epididymal spermatozoa provide an additional source of male gametes available for cryopreservation. While this procedure is feasible, the survival rate and motility of epididymal cat spermatozoa are both low after thawing. Cryopreservation is known to induce oxidative stress in spermatozoa, with mitochondria and the plasma membrane being the two major generation sites, and an imbalanced presence of free radicals is a possible cause for this low survival rate. Different antioxidants have been tested before for their effect on cryopreserved cat spermatozoa quality, with varying results. Here, we used Mito-Tempo, which is a synthetic mitochondria-targeted antioxidant and a specific scavenger of the mitochondrial superoxide system. By supplementing Mito-Tempo with the freezing extender, we aimed to improve the sperm quality of frozen-thawed cat epididymal spermatozoa.

**Methods:**

Epididymal spermatozoa obtained from twelve tomcats were assessed for motility and concentration. Prior to freezing, samples were diluted in TRIS buffered extender with egg yolk and glycerol and divided into five aliquots supplemented with 0 (control), 0.5, 5, 50, and 1005M of Mito-Tempo. After thawing, sperm motility, concentration, morphology, plasma membrane integrity, acrosome integrity, and mitochondrial membrane potential were evaluated. A Friedman rank sum test with a Bonferroni post-hoc test was used to determine statistical in-between group differences in post-thaw semen parameters.

**Results and discussion:**

The results indicated a slight improvement in acrosome integrity across all groups that were supplemented with Mito-Tempo, with the group that received 55M of Mito-Tempo showing the greatest improvement [(median of 67.99%, IQR of 5.55) compared to the control group (median of 65.33%, IQR of 7.75; *P* = 0.05)]. For all other sperm parameters, no significant differences (*P* > 0.05) were detected between different Mito-Tempo concentrations. These findings highlight the protective effect of Mito-Tempo on acrosome integrity and suggest that 55M is the most effective concentration for maintaining acrosome integrity. Since Mito-Tempo has shown a positive effect on multiple sperm parameters in other species, such as men, boars, roosters, rams, and bulls, we need to conclude that species-specificity may play a role here.

## 1. Introduction

Over 60% of the Felidae are classified as endangered and vulnerable or near threatened by the International Union for the Conservation of Nature (1). The fragmentation of Felidae populations into smaller, isolated groups is, among other factors, leading to an increased risk of extinction (2–4). This fragmentation is reducing gene flow, leading to a reduction in genetic diversity that results in a decrease in fertility (2, 5). Studies have shown that limited genetic variability is associated with higher production of malformed spermatozoa (5). For this reason, it is extremely important to encourage genetic variability by preserving gametes from a wider number of animals. To this aim, deceased animals can represent a convenient source for the collection of gametes to enrich genetic banks. The optimization of collection and preservation protocols is the key to support endangered species preservation and the domestic cat (*Felis catus*) represents an excellent model, providing an accessible and more abundant source of gametes.

Cryopreservation is widely used to preserve animal and human gametes (6), but the process can result in significant damage to various aspects of sperm parameters, such as the plasma membrane integrity, acrosome integrity, sperm motility, and DNA integrity (7). This degradation is primarily caused by the formation of free radicals. Free radicals can be categorized into reactive oxygen species (ROS), reactive nitrogen species, and other non-radical reactive species (6). Among these, the most commonly encountered free radicals are those belonging to the ROS family. An imbalance in the oxidant-antioxidant system caused by an overproduction of oxidants results in oxidative stress. The resulting oxidative stress can lead to lipid peroxidation, where polyunsaturated fatty acids in the plasma membrane are attacked by ROS, resulting in the formation of lipid peroxide molecules that cause physical and functional alterations to the plasma membrane, such as increased permeability, decreased fluidity, and changes in membrane protein function (6). Reactive Oxygen Species that enter the spermatozoa pose a significant threat to the genetic material. They cause destruction of the mitochondrial DNA, leading to a reduction in intracellular ATP production (8, 9). This reduction is affecting both sperm function and motility (10).

**Figure 1:**
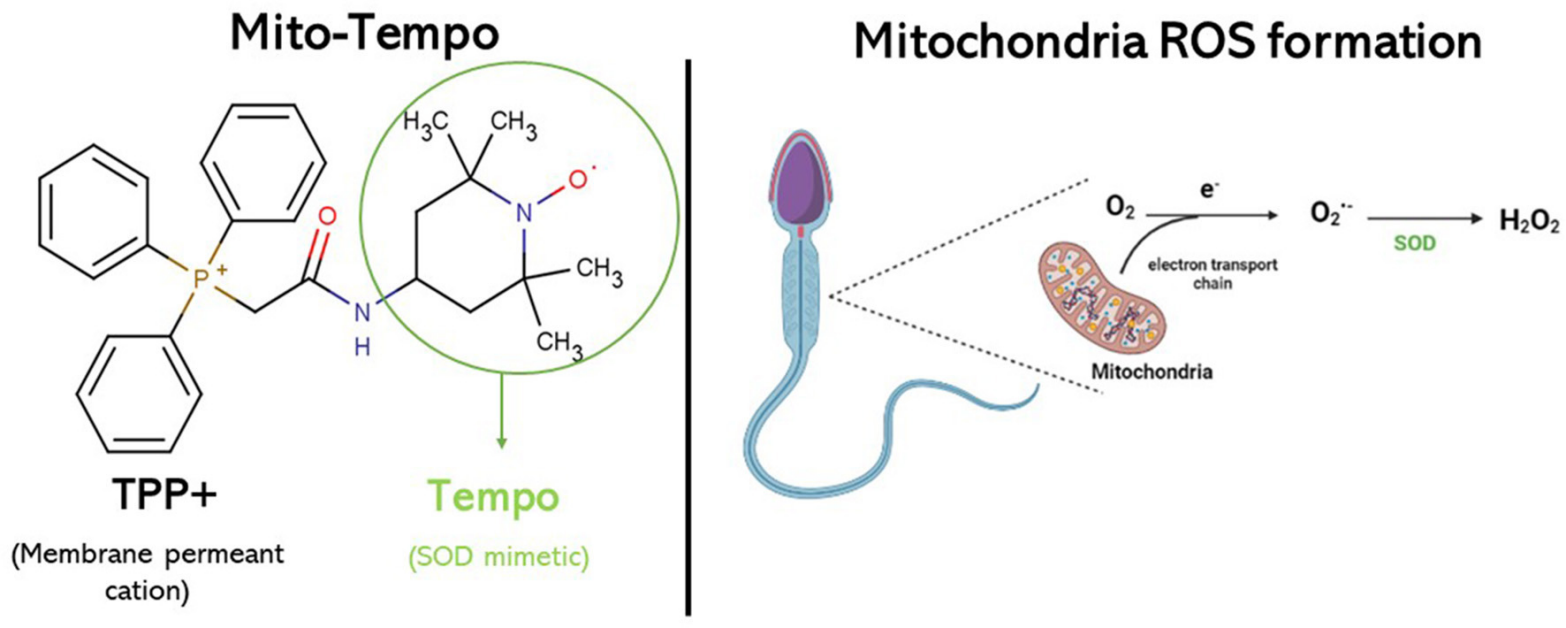
Mito-Tempo molecular formula. Acting as SOD mimetic, it can decrease ROS generation in the inner mitochondrial membrane.

Antioxidants employed in cryopreservation extenders may serve as a safeguard against the formation of ROS and the onset of oxidative stress. These antioxidants can be classified into enzymatic activity antioxidants (such as glutathione peroxidase, superoxide dismutase (SOD), and catalase) or non-enzymatic activity antioxidants (such as vitamin C, vitamin E, vitamin B12, melatonine, resveratrol, and glutathione) (11). For this reason, several antioxidants have been tested as additives to the freezing extender in different species to improve sperm cryosurvival (12, 13). Mito-Tempo (MT) is a synthesized mitochondria-targeted antioxidant derived from piperidine nitroxide, TEMPO, conjugated with a lipophilic triphenylphosphonium cation (TPP+) and functions as a specific scavenger of mitochondrial superoxide. TEMPO works as a superoxide dismutase mimetic in the catalytic cycle of superoxide (Figure 1). TPP^+^ is a membrane permeant cation that, driven by membrane potential, rapidly passes the lipid membrane and massively accumulates in energized mitochondria. The combination of TEMPO and TPP^+^ creates a chemical with superoxide scavenging properties which specifically targets the mitochondria (8, 9, 14).

The effect of MT supplementation to semen extender has been investigated in various species such as humans (15, 16), boars (17), roosters (18, 19), rams (20), and bulls (21, 22). In these studies, sperm quality was revamped upon supplementation of MT when compared to the control group. To our knowledge, the effect of MT supplementation to semen extender has never been investigated in felids. The present research aimed therefore to assess the impact of this specific antioxidant on the post-thaw quality of feline epididymal spermatozoa.

## 2. Materials and methods

All products were purchased from Sigma (Sigma, St. Louis, USA) except if stated otherwise.

### 2.1. Animal samples

Testes and epididymides from twelve tomcats were collected from multiple first-line veterinary clinics across Wroclaw, Poland between April and May 2022. All tomcats were clinically healthy stray cats and underwent elective orchiectomy in a trap, neuter and release programme. After collection, samples were immediately placed into 50 mL falcon tubes containing 0.9% NaCl and transported inside a portable fridge (4°C with controlled temperature system) to the laboratory of the Faculty of Veterinary Medicine, Wroclaw University of Environmental and Life Science. Samples were processed within 1–4 h following orchiectomy. Briefly, after being removed from the physiological saline solution, the samples were washed with phosphate buffer saline (PBS; P4474) and each epididymis was carefully dissected away from the testis. Epididymal mincing was performed for spermatozoa collection. Specifically, the cauda from both epididymides, along with the ductus deferens, were placed into a Petri dish containing 5 mL of semen collection medium [Ham's F-10 medium (N6013) supplemented with 2 mM of L-glutamine (G7513), and 5% of Fetal Bovine Serum (F9665)]. A sterile scalpel blade was used to perform multiple cuts, avoiding blood vessels, on both the cauda and the ductus deferens, allowing the spermatozoa to swim out into the semen collection medium. After 10 mins of incubation at 37°C, the semen collection medium containing the spermatozoa was collected, filtered (CellTrics 30 mm, Partec) and placed into a pre-warmed 15 mL falcon tube.

### 2.2. Pre-freezing sperm assessment

#### 2.2.1. Sperm motility

Sperm motility was evaluated immediately following collection of the spermatozoa. Briefly, 10 μL of the sperm suspension was placed on a pre-warmed microscopic slide, covered with a pre-warmed glass cover slip, and assessed subjectively by two equally experienced operators under a phase-contrast microscope equipped with a warming stage (37°C) (Nikon Eclipse E200). Total sperm motility was evaluated under five different fields by both operators and the mean was calculated and recorded.

#### 2.2.2. Sperm concentration

Sperm concentration was measured using a Thoma counting chamber. For this procedure, 10 μL of the sperm suspension were diluted into 190 μL of water. After delicately mixing the suspension, both grids of the Thoma chamber were filled with 10 μL of the suspension. Spermatozoa were counted using a phase-contrast microscope (magnification x40) (Nikon Eclipse E200) and the concentration was calculated.

### 2.3. Cryopreservation and thawing

Each sperm sample was centrifuged at 620 × *g* for 5 mins at 22°C (23). The supernatant was removed, and the pellet was resuspended into semen extender I, containing 3% glycerol and 20% egg yolk in Tris buffer [3.025 g Tris(hydroxymethyl)aminomethane (T6066), 1.7 g citric acid (C7129), 1.25 g fructose (F3510), and 0.1 g streptomycin (S9137) in 100 ml distilled water] to reach a concentration of 16 × 10^6^ spermatozoa/ml (24). The extended sample was then divided into four equal volumes and placed into 2 ml Eppendorf tubes (Eppendorf, Germany).

Each aliquot was supplemented with a specific concentration of Mito-Tempo (MT; SML0737) (Group A control: no MT; Group B: 5 μM MT; Group C: 50 μM MT; and Group D: 100 μM MT). The MT volume to reach the desired concentration in each aliquot was calculated and half of it was immediately added into each Eppendorf tube. Afterwards, samples were placed in a warm water bath (37°C) and cooled to 4°C. After 90 mins, the second semen extender (extender II containing 7% glycerol, 1% Equex paste, and 20% egg yolk in Tris buffer) was added alongside the other half of the calculated amount of MT to reach an final concentration of 8 × 10^6^ spermatozoa/ml (24). The samples were then directly loaded into precooled 0.25 mL straws, placed 5 cm above the surface of liquid nitrogen for 10 mins before being plunged into liquid nitrogen (23). Straws were then stored at-−196°C for at least 3 days before further analyses (23).

Thawing was obtained by submerging the straws in a 37°C warm water bath for 30 secs and post-thaw sperm parameters were immediately evaluated (23).

### 2.4. Post-thaw sperm evaluation

#### 2.4.1. Sperm motility and morphology

Sperm motility was evaluated as previously described for fresh samples. Sperm morphology was assessed after eosin nigrosin staining. Briefly, 10 μL of eosin nigrosin solution (v:v) were added to 10 μL of thawed sperm suspension and smeared onto a microscopic slide. After the slide was air dried, two hundred sperm cells were assessed under a bright-field microscopy (Olympus BX51TF, Tokyo, Japan) at 1,000x magnification under oil immersion. The percentages of morphologically normal sperm cells and sperm cells with abnormal heads, abnormal tails, proximal cytoplasmic droplets, and distal cytoplasmic droplets were then recorded.

#### 2.4.2. Flow cytometer analysis

The post-thaw semen samples were diluted with TRIS buffer (3.02% (w/v) TRIS, 1.35% (w/v) citric acid, 1.25% (w / v) fructose, in distilled water; pH 6.5, all reagents purchased from Merck, Poland) to obtain a concentration of 5 × 10^6^ spermatozoa/mL. Each diluted sample was then divided into three aliquots of 300 μL each to assess plasma membrane integrity, acrosome integrity, and mitochondrial activity by flowcytometry Guava EasyCyte 5 (Merck KGaA, Darmstadt, Germany) cytometer. The fluorescent probes were excited by an Argon ion 488-nm laser. Detection of green fluorescence was set with an FL1 band-pass filter (525 nm / 30 nm), orange fluorescence was measured using FL2 filter (583/26 nm) and red fluorescence was measured using an FL3 filter (695/50 nm). The non-sperm events were gated out based on scatter properties and excluded from the analysis. A total of 10,000 events were analyzed per parameter for each sample. Gametes acquisitions were analyzed with the GuavaSoft™ 3.1.1 software (Merck KGaA, Darmstadt, Germany).

##### 2.4.2.1. Plasma membrane integrity

Plasma membrane integrity was assessed using SYBR-14 and propidium iodide (PI) (Live/Dead Sperm Viability Kit; L7011, Life Technologies Ltd, Carlsbad, CA, USA) according to Prochowska et al. (23). SYBR-14 is a membrane permeable fluorescent dye that binds to DNA in live sperm cells and emits green fluorescence. SYBR-14 is used in combination with propidium iodide (PI), a DNA-specific stain that cannot enter the intact plasma membrane, as a dead-marker counterstain (25). Briefly, 5 μL of 0.02 mM of SYBR-14 was added to 300 μL of sperm suspension and incubated at room temperature in the dark for 10 mins. Afterwards, 1.8 μL of 2.4 mM PI was added and the sample was immediately analyzed by flow cytometry. The percentage of sperm cells with an intact plasma membrane was recorded.

##### 2.4.2.2. Acrosome integrity

Acrosome status was evaluated using lectin PNA (PNA from *Arachis hypogaea*, Alexa Fluor 488 conjugate; L21409, LifeTechnologies Ltd, Carlsbad, CA, USA) and PI according to Prochowska et al. (23). PNA is a lectin conjugated with Alexa Fluor 488 (green-fluorescent dye) as a fluorescent probe. It specifically binds to β-galactose on the outer acrosomal membrane of sperm cells (26). Briefly, 6 μL of 0.1 mg/mL PNA was added to 300 μL of sperm suspension and incubated at room temperature in the dark for 5 mins. Afterwards, the sample was centrifuged at 620 × *g* for 5 mins and the pellet was resuspended with 300 μL of TRIS. Finally, 1.8 μL of 2.4 mM PI was added and the sample was immediately analyzed by flow cytometry. The percentage of sperm cells with an intact acrosome was recorded.

##### 2.4.2.3. Sperm mitochondrial membrane potential

Evaluation of the sperm mitochondrial membrane potential was performed using JC-1 staining (T3168, Life Technologies Ltd, Carlsbad, CA, USA) according to Prochowska et al. (23). JC-1 is a lipophilic cationic fluorescent dye that specifically targets mitochondria. When the mitochondrial potential is low, JC-1 emits green fluorescence in its monomer form, while in high mitochondrial potential, JC-1 emits orange fluorescence in its J-aggregate form (23). Briefly, 0.4 μL of JC-1 solution (2 mg/ml JC-1 in DMSO) was added to 300 μL of the semen sample. Samples were incubated at 37°C in the dark for 20 mins. Afterwards, 1.8 μL of 2.4 mM PI was added and the sample was immediately analyzed by flow cytometry. The percentage of live sperm cells with high mitochondrial activity was recorded.

### 2.5. Statistical analysis

The statistical analysis was conducted using R software version 4.1.2 (R Foundation for Statistical Computing, Vienna, Austria). Normality of the fresh and post-thaw semen parameters was assessed using Shapiro-Wilk test (*P* ≤ 0.05). Friedman rank sum test with Bonferroni post-hoc test was used to determine statistical in-between group differences in post-thaw semen parameters. Specifically, differences in the amounts of motile spermatozoa (%), morphologically normal spermatozoa (%), abnormal sperm heads (%), abnormal sperm tails (%), proximal (%) and distal cytoplasmatic droplets (%), plasma membrane integrity (%), acrosome integrity (%), and mitochondrial membrane potential (%) were assessed. Significance was considered for *p*-values ≤ 0.05.

## 3. Results

Acrosome integrity was significantly improved in all samples treated with MT in comparison to untreated samples. Specifically, group B (5 μM of MT) appeared to exert the most protective effect on acrosome integrity compared to the control group (median 67.90%, IQR 5.55 and median 65.33%, IQR 7.75, respectively; *P* = 0.05), whereas group C and group D only showed a tendency to improve acrosome integrity in comparison to the control group (*P* = 0.07 for both concentrations). No significant differences in the percentages of motile, morphologically normal spermatozoa, or morphologically abnormal sperm cells were found between the different groups investigated. Nor were any differences found between the investigated groups in terms of plasma membrane integrity and mitochondrial membrane potential. Results (median, IQR, and overall p-values) are reported in Table 1.

**Table 1:** Effect of Mito-Tempo on post-thaw sperm parameters in treatment groups.

	**Group A (control)**		**Group B (5** μ**M)**		**Group C (50** μ**M)**		**Group D (100** μ**M)**		**Overall *P*-value**
	**Median**	**IQR**	**Median**	**IQR**	**Median**	**IQR**	**Median**	**IQR**	
Motile spermatozoa (%)	33.75	25.00	27.50	11.87	30.00	23.12	30.00	16.25	0.84
Morphologically normal spermatozoa (%)	61.00	16.75	63.00	23.75	65.00	17.25	61.00	16.00	0.77
Abnormal sperm head (%)	10.50	7.50	10.50	7.50	9.00	17.00	10.00	4.50	0.91
Abnormal sperm tail (%)	15.50	20.00	15.00	18.00	17.50	14.00	17.00	15.00	0.66
Proximal cytoplasmic droplet (%)	4.00	2.25	4.50	3.25	4.00	1.75	3.00	3.25	0.10
Distal cytoplasmic droplet (%)	5.00	4.50	6.50	6.25	5.00	3.50	4.50	1.75	0.13
Plasma membrane integrity (%)	42.45	15.84	35.39	12.74	37.42	15.42	41.68	12.39	0.37
Acrosome integrity (%)	65.33^a^	7.75	67.90^b^	5.55	66.67	6.55	66.46	8.17	0.07
Mitochondrial membrane potential (%)	30.25	36.52	25.94	25.34	57.95	69.78	23.82	21.16	0.99

a, b Indicate significant differences (P ≤ 0.05).

## 4. Discussion

In the present study, we found a small increase in acrosome integrity in frozen-thawed cat epididymal semen supplemented with 5 μM of MT during freezing. All the other investigated parameters, such as sperm motility, morphology, plasma membrane integrity, and mitochondrial membrane potential, were not significantly improved. This finding was in contrast with previous studies conducted in other species, which demonstrated improvement in sperm parameters such as motility, membrane functionality, mitochondrial active potential, acrosome integrity, and viability, as well as a decrease in lipid peroxidation and DNA fragmentation (18–21). It should be noted that the assessment of sperm motility in this study was conducted subjectively. Although two operators with equal experience evaluated motility in five different fields and calculated the mean after counting spermatozoa, a significant limitation arises from the absence of more advanced techniques such as computer-assisted sperm analysis (CASA). Subjective motility assessment may not adequately capture subtle variations in motility patterns or minor changes over time, which can restrict the detection of more nuanced alterations in sperm motility. Furthermore, CASA offers the opportunity to evaluate additional characteristics of sperm kinematics that could be influenced by the supplementation of Mito-Tempo. For instance, higher straight-line velocity and amplitude of lateral head displacement are frequently associated with capacitation. Considering that Mito-Tempo exhibited a mild enhancement in acrosome integrity, it would be beneficial to assess these parameters by CASA as well to gain a more comprehensive understanding of the effects of Mito-Tempo supplementation.

Although the exact cause for this disparity remains unclear, Len et al. (27) has suggested before that antioxidants might act in a species-specific manner. For instance, the supplementation of 200 IU/ml catalase (CAT), a hydrogen peroxide targeted enzymatic antioxidant, to Tris egg yolk glycerol (TEY) extender reduced the motility of feline sperm cells (28), although supplementation of CAT at the same concentration increased the same parameter in cryopreserved bovine spermatozoa (29). Unexpectedly, adding double the concentration of CAT (400 IU/ml) did not improve the motility, viability, or acrosomal integrity of frozen-thawed cat spermatozoa (30). Therefore, an interspecies difference between cats and previously studied species that were supplemented with MT (15–22) may be a potential explanation. Epididymal spermatozoa are believed to have lower levels of antioxidant enzymes due to the lack of exposure to seminal plasma, making them more vulnerable to oxidative stress. However, in the case of cat semen, there is conflicting evidence as it is thought to be more resilient to lipid peroxidation (23). Various studies have supported this claim. In comparison, post-thaw epididymal cat spermatozoa did not experience an increase in lipid peroxidation after 6 h of incubation at 37°C (31), while frozen-thawed human spermatozoa showed increased lipid peroxidation after being incubated for 15 to 60 mins at the same temperature (32). Additionally, cold storage of equine spermatozoa for 48 h led to a significant increase in lipid peroxidation (33), whereas cat epididymal sperm stored at 5°C maintained high quality for up to 48 h of cooling and were not exposed to oxidative stress until after 72 h of cooling (34). It can be hypothesized that high levels of endogenous antioxidant activity may be present in cat spermatozoa and/or epididymal fluid that may neutralize excessive ROS concentrations during semen processing and cryopreservation. Indirect evidence for such endogenous antioxidant activity was demonstrated by Thuwanut et al. (31) who found that lipid peroxidation was only detected after 6 h post-thaw incubation with lipid peroxidation promoter [100 mM ferrous sulfate (FeSO4)] in epididymal cat spermatozoa.

Our study detected a mild protective effect of MT on acrosome integrity in epididymal cat spermatozoa after thawing when supplemented with a 5 μM concentration. Cryocapacitation, a capacitation-like change, occurs in spermatozoa during the freeze-thaw process, but its mechanism is not wellunderstood (35). Mito-Tempo is an antioxidant that acts as a mimetic of SOD, a substance that helps to preserve normal acrosome integrity and prevents premature hyperactivation and capacitation by superoxide radicals before ejaculation (36). Although the enhancement of acrosome integrity in our study was not substantial, it is an important factor that correlates directly with fertility rate. Compared to sperm motility and morphology, previous research has indicated that assessing the integrity of the plasma membrane and acrosome is a more dependable indicator for predicting *in vitro* fertility rate (37, 38). Verstegen et al. (39) reported that high levels of sperm with acrosome defects are associated with fertilization difficulties. Also, Tanghe et al. (40) demonstrated a moderate correlation between acrosome integrity and pronuclei formation after *in vitro* fertilization in bovine.

Life is a balance of opposing forces such as oxidant and antioxidant. A proper balance between these two elements is maintained through equalization, with any disequilibrium leading to potential damage. Thus antioxidants should only be used as a supplement when oxidant overproduction is expected or/and when antioxidant defense system is weakened. Consequently when antioxidant is used without a rationale, the supplementation of these additives to the semen extender might even be detrimental (41). An example of this phenomenon is the effect of catalase supplementation to the extender of chilled ram semen. Concentrations over 200 U/mL decreased the sperm quality, whereas lower concentrations have a positive effect (42). The choice of testing concentrations ranging from 0 to 100 μM was based on research on other species. Significant results were obtained upon supplementation of 5 μM and/or 50 μM of MT to the extender in several species (15, 17–22), whereas another study reported an improvement in human semen parameters, and an enhancement of antioxidant enzymes activity upon supplementation of 10 μM and 100 μM of MT (16). Nevertheless, none of the concentration chosen in the present study suggested that increasing the concentration would be neither beneficial nor detrimental for feline sperm cells. Therefore, combining two antioxidants with different functional properties, may have an additive positive effect, mitigating the cryo-stress instead of increasing the concentration of an antioxidant. For instance, when catalase and SOD were both added to an extender for boar semen, a greater improvement in post-thaw sperm parameters was achieved compared to the supplementation of either of them alone (43). Mito-tempo is a SOD mimetic antioxidant that can be targeted to mitochondria in protecting against the selective mitochondrial oxidant stress, scavenging the superoxide anion (14). On the other hand, glutathione peroxidase can then breakdown hydrogen peroxide into oxygen and water. Since the positive effect of glutathione peroxidase on feline spermatozoa has been proven (35), it might be worth investigating if combining MT or SOD with glutathione peroxidase could further enhance their protective performance on feline sperm parameters.

## 5. Conclusion

Our results showed that Mito-Tempo supplementation improved acrosome integrity with no positive effect on all other semen parameters investigated in this study. Previous studies have not examined the use of Mito-Tempo as a supplement to semen extenders for both ejaculated and epididymal spermatozoa in cats, so it is not possible to compare our findings to other similar studies. However, in recent years, the effect of supplementing various antioxidants to freezing extenders on sperm quality has been widely studied in various species, with inconsistent improvement of sperm quality amongst species, including felids (13, 27, 28, 44). Further research should be performed to investigate another and/or larger feline population. In addition to investigate the effect of MT on ROS production which may reveal potential combinations of MT with antioxidants.

## Data availability statement

The raw data supporting the conclusions of this article will be made available by the authors, without undue reservation.

## Ethics statement

Ethical review and approval was not required for the animal study because the study analyzed epididymal spermatozoa from cats that had undergone castration either as a part of a spay program or based on the owner's request. The castration was not performed for the purpose of the study, but the samples were utilized to prevent their wastage. It is important to note that none of the cryopreserved sperm was used for insemination or *in vitro* fertilization. Instead, all post-thaw samples were thawed and analyzed to evaluate the effect of Mito-Tempo supplementation on various sperm parameters.

## Author contributions

HA and AV: conceptualization. HA, SP, and LV: methodology and investigation. PB: statistical analysis. HA and SP: resources. HA and PB: writing—original draft preparation and visualization. SP, GD, and AV: writing—review and editing. SP, AV, and WN: supervision. AV and WN: funding acquisition. All authors have read and agreed to the published version of the manuscript.
